# Modification of the genome topology network and its application to the comparison of group B *Streptococcus* genomes

**DOI:** 10.1186/s12864-019-6234-8

**Published:** 2019-11-21

**Authors:** Xiao Deng, Xuechao Zhao, Yuan Liang, Liang Zhang, Jianping Jiang, Guoping Zhao, Yan Zhou

**Affiliations:** 1 Institutes of Biology and Medical Sciences, Medical College of Soochow University, Suzhou, 215123 China; 2 State Key Laboratory of Genetic Engineering, School of Life Sciences, Fudan University, Shanghai, 200433 China; 3 Shanghai-MOST Key Laboratory of Health and Disease Genomics, Chinese National Human Genome Center at Shanghai, Shanghai, 201203 China; 4 SJTU-Yale Joint Center for Biostatistics, Shanghai Jiaotong University, Shanghai, 200240 China

**Keywords:** Genome topology network, Genomes, Phylogenetics, Group B *Streptococcus*, Clusters of orthologous groups (COG)

## Abstract

**Background:**

The genome topology network (GTN) is a new approach for studying the phylogenetics of bacterial genomes by analysing their gene order. The previous GTN tool gives a phylogenetic tree and calculate the different degrees (DD) of various adjacent gene families with complete genome data, but it is limited to the gene family level.

**Result:**

In this study, we collected 51 published complete and draft group B *Streptococcus* (GBS) genomes from the NCBI database as the case study data. The phylogenetic tree obtained from the GTN method assigned the genomes into six main clades. Compared with single nucleotide polymorphism (SNP)-based method, the GTN method exhibited a higher resolution in two clades. The gene families located at unique node connections in these clades were associated with the clusters of orthologous groups (COG) functional categories of “[G] Carbohydrate transport and metabolism,”, “[L] Replication, recombination, and repair” and “[J] translation, ribosomal structure and biogenesis”. Thus, these genes were the major factors affecting the differentiation of these six clades in the phylogenetic tree obtained from the GTN.

**Conclusion:**

The modified GTN analyzes draft genomic data and exhibits greater functionality than the previous version. The gene family clustering algorithm embedded in the GTN tool is optimized by introducing the Markov cluster algorithm (MCL) tool to assign genes to functional gene families. A bootstrap test is performed to verify the credibility of the clades when allowing users to adjust the relationships of the clades accordingly. The GTN tool gives additional evolutionary information that is a useful complement to the SNP-based method. Information on the differences in the connections between a gene and its adjacent genes in species or clades is easily obtained. The modified GTN tool can be downloaded from https://github.com/0232/Genome_topology_network

## Background

### Gene order can serve as evidence for evolutionary research

The development of gene sequencing technology has led to increases in the amount of available genomic sequencing data and the number of evolutionary analysis tools. The majority of the methods for phylogenetic analysis are based on nucleotide sequence alignment and SNP analysis. These methods include the RAxML [1] and IQ-TREE [2] tools, which are phylogenetic analysis tools employing the maximum likelihood approach. Some pangenome analysis tools, such as PGAP [3] and panX [4], are also available for phylogenetic analysis through the assessment of gene losses or gains or SNP mutations in core genes.

Gene order is substantial information arising from the evolutionary study of yeast genomes [5] and the mitochondrial genomes of fungi [6] and plants [7]. Some conserved gene orders and contents affect functional protein interactions [8] or speciation [9]. Yang YF et al. [10] successfully predicted gene order in budding yeast on the basis of a genetic interaction network. In addition to SNP-based analysis, gene order conservation is an effective measure for the study of bacterial evolution [11]. Dandekar et al. [12] studied the structure of the tryptophan operon in different bacterial genomes and found that the order of homologous genes could be classified. The reconstruction of deep evolutionary histories by analysing molecular sequence data is always difficult, but differences in gene order allow the determination of genomic evolutionary events such as gene recombination, indels, and duplications [13].

### The GTN was developed on the basis of different gene orders in different genomes

**Fig. 1:**
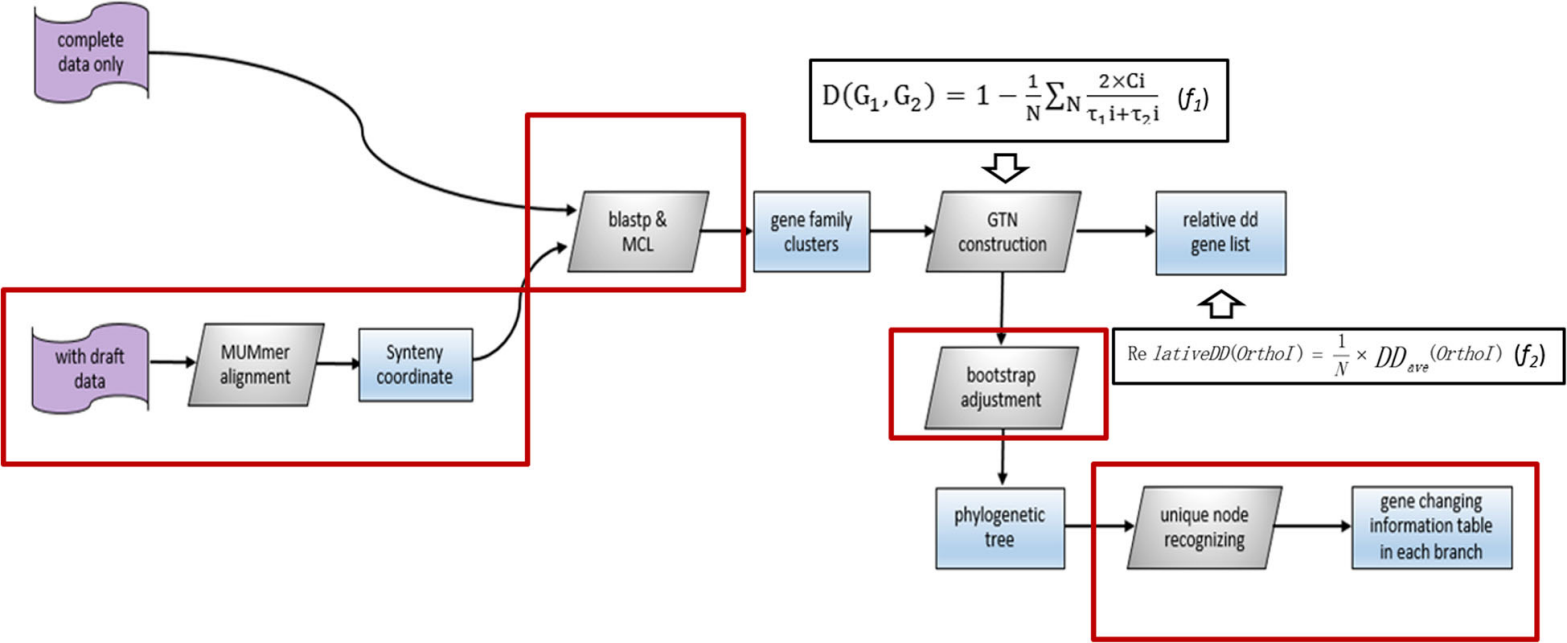
Work flow of the new GTN version. The GTN calculates the whole genome region when only complete genomes are included or the common synteny block regions when draft data are included. Then, the GTN assigns genes to different gene families and calculates the relative DD value and the evolutionary distance on the basis of the gene family assignment. After obtaining the phylogenetic tree, the GTN determines all genes at unique node connections. The steps in red boxes are the modifications included in this GTN version. In *f*_*1*_, D (G1, G2) represents the evolutionary distance between genomes 1 and 2, *N* represents the number of nonredundant families among the total gene families, Ci represents the number of common adjacent gene families to *orthoi*, τ_1_i represents the number of adjacent gene families to *orthoI* in genome 1 and can be regarded as the number of connections constituting the *orthoI* network in genome 1, and τ_2_i represents the number of adjacent gene families to *orthoI* in genome 2. *N* in *f*_*2*_ represents the number of genes in the gene family

We previously developed the first version of the genome topology network (GTN) [14], which is a new approach for studying closely related bacterial genomes by analysing gene order in complete genomes. The primary function of the first GTN version is to provide a phylogenetic tree on the basis of an evolutionary distance matrix calculated using the formula provided in Fig. 1 (*f*_*1*_) [14]. This formula indicates that the evolutionary distance between two genomes is affected by different gene family connections, which are known as “edges” in the first GTN version. The analysis performed by the first GTN version is focussed on the gene family level and not on the gene level. Complete genomes are the only data type that the first GTN version can analyze. However, the most widely used databases include extremely large numbers of draft genomes, exceeding number of available complete genomes. Therefore, more biological information can be obtained if draft genomic data are added to the calculation compared to that obtained using the first GTN version. In this study, the four following improvements were implemented in the new GTN version:

1. Draft genome data can be calculated by analysing the common synteny blocks of all genomes. Compared with the data for a complete genome, draft genome data may lack some sequences. Our strategy is to use the MUMmer [15] tool to detect the common synteny blocks that exist in all draft and complete genomes to calculate evolutionary distance.
2. The MCL tool [16], which is an algorithm that is used in many authoritative clustering tools (e.g., orthoMCL [17] and Get_homologues [18]), was applied in the GTN to cluster genes into Clusters of Orthologous Groups (COGs) families. COG is a database consisting of functional annotation of gene classification. It was calculated by comparing predicted and known proteins in all completely sequenced microbial genomes to infer sets of orthologues [19]. Each COG family is annotated functional characteristics so that we can obtain the cluster functional annotation when it is assigned to a COG family.
3. A bootstrap test was used to adjust the phylogenetic tree and improve the GTN’s robustness.
4. The new GTN version can easily find genes at the unique node connections of genomes or clades, thereby demonstrating the most notable modifications. These genes can be utilized to explore the genes involved in genome differentiation. This function is lacking in the first GTN version.

### Details of the introduction of the distance calculation in GTN

The new GTN workflow is shown in Fig. 1. In formula *f*_*1,*_ which is cited from our first version of the GTN [14], D(G1, G2) represents the distance between Genome1 and Genome2, N represents the total number of orthologues (COG families in this paper, or ‘nodes’ in the first version of the GTN), Ci represents the number of orthologues adjacent to *orthoi* (gene connections in this paper, or ‘edges’ in the first version of the GTN) in both Genome1 and Genome2, and τ_1_i and τ_2_i represent the number of orthologues adjacent to *orthoi* in Genome1 and Genome2, respectively.

Formula *f*_*1*_ is used to calculate the evolutionary distance between two genomes. A distance matrix file that consists of all evolutionary distances is obtained by the GTN to draw a phylogenetic tree using the neighbour-joining (NJ) method, which is one of the most common phylogenetic algorithms [20]. Formula *f*_*1*_ suggests that evolutionary distance is related to all adjacent gene families of all gene families, thereby essentially reflecting the gene order in genomes. A gene connection can be defined as two adjacent genes according to the coordinates in the general feature format (GFF) file. The GTN consists of all gene connections in this genome.

**Fig. 2:**
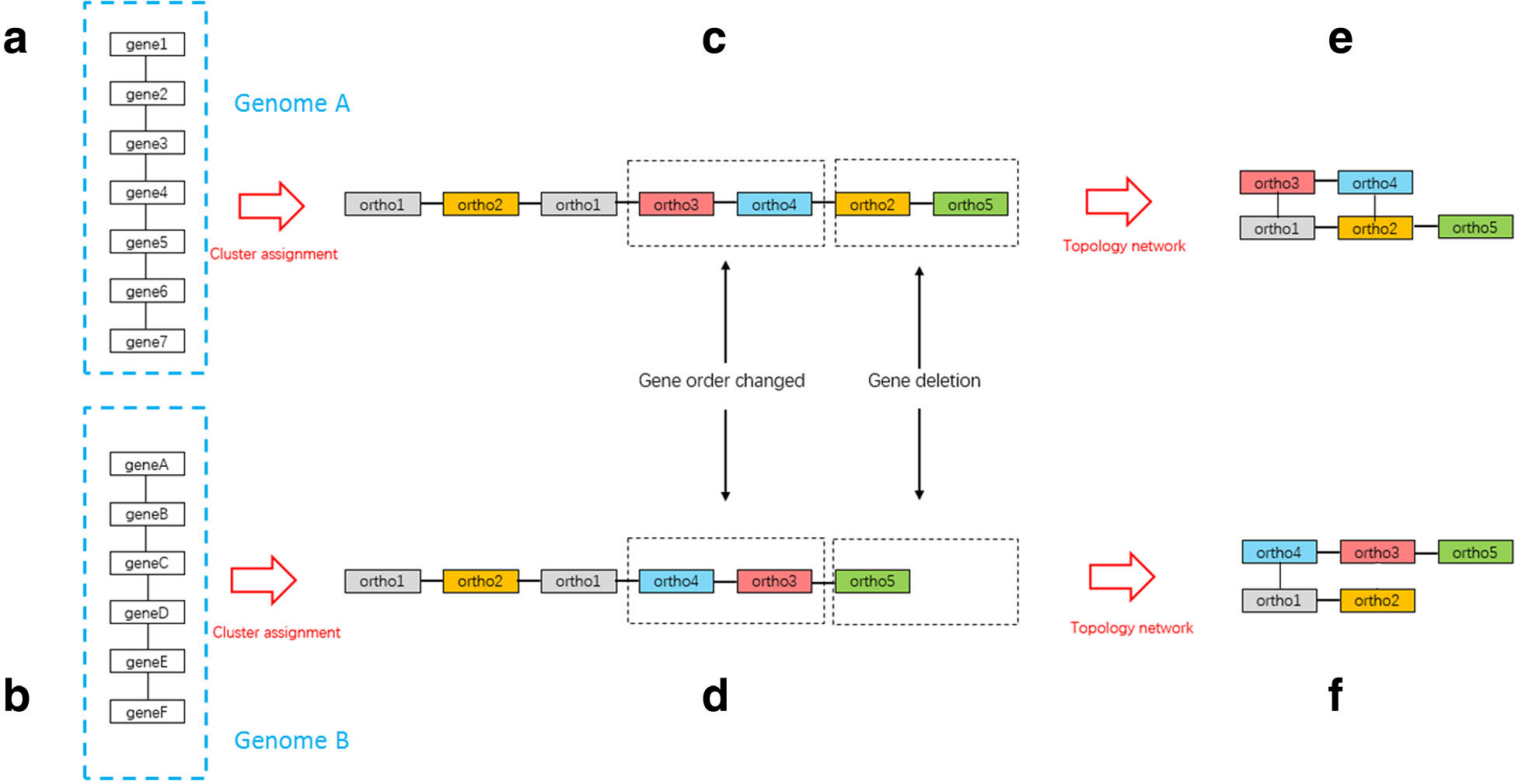
Example of the topology networks of two genomes. **a** Location information for genes in the assumed genome A on the basis of the GFF file. **b** Location information for genes in the assumed genome B on the basis of the GFF file. **c** Gene order of orthologous genes in the assumed genome A after clustering. **d** Gene order of orthologous genes in the assumed genome **b** after clustering. Compared with that in genome **a**, the gene order of *ortho3* and *ortho4* is changed, and an *ortho2* gene is deleted. E. Topology network of genome. **a**. **f** Topology network of genome **b**

As shown in Fig. 2 as an example, gene information is obtained from GFF files, and genes are then assigned to clusters of orthologues to obtain the gene order of each genome. The gene connections in genome A can be described as follows: ortho1-ortho2, ortho1-ortho3, ortho2-ortho4, ortho2-ortho5, and ortho3-ortho4 (Fig. 2c). The topology network of genome A can be described as the network in Fig. 2e. The gene order of ortho3 and ortho4 according to the GFF file was changed in genome B, and an ortho2 gene was deleted. The gene connections in genome B can be described as follows: ortho1-ortho2, ortho1-ortho4, ortho3-ortho4, and ortho3-ortho5 (Fig. 2d). The topology network of genome B is shown in Fig. 2f.

According to *f*_*1*_ in Fig. 1, if these genomes have the same number of gene families and each gene family shows the same connection to its adjacent gene families, then the evolutionary distance between two genomes will be 0. This result suggests that the evolutionary distance calculated by the GTN is based on different gene connections. A gene connection that only exists in one genome and is absent in its reference genome is defined as a unique node connection in the GTN. These unique node connections can alter gene order and affect genome or clade separation, and they reflect evolutionary events in a genome such as gene duplications, gene insertions and deletions, and gene recombination. As shown in Fig. 2, three unique node connections are present in genome A (i.e., ortho1-ortho3, ortho2-ortho4, and ortho2-ortho5), and two unique node connections are present in genome B (i.e., ortho1-ortho4 and ortho3-ortho5). In the GTN, these five unique node connections render these two genomes different.

### Details of the introduction of different degree values

The different degree (DD) values of a gene family provided by the GTN represent the tendency of the gene family to change its connections. The DD value can be described as follows [14]:

$$ {\mathrm{DD}}_{\mathrm{ave}}(orthoI)=\mathrm{AVE}{\left[\mathrm{ROUNDDOWN}\left(\sum |\mathrm{degree}{\left( orthoI, orthoi\right)}_{\mathrm{p}}-\mathrm{degree}{\left( orthoI, orthoi\right)}_{\mathrm{q}}|\right)\right]}_{\mathrm{p}\ne \mathrm{q}} $$

DD_p,q_(*orthoI*) represents the DD value of orthoI in genomes p and q, and *orthoi* represents all orthologues adjacent to *orthoI* in genomes p and q.

If the DD value of a gene family is 0, then this gene family exhibits the same connections to its adjacent gene families in all GTNs. Considering that the number of genes in every gene family is different, the greater the gene number in a gene family, the greater the possibility that this family exhibits differences in adjacency. Thus, the relative DD, which divides DD values into the number of genes in the gene family (‘N’ in *f*_*2*_), must be used to evaluate the connection-changing tendency of its genes (*f*_*2*_ in Fig. 1).

#### *Streptococcus agalactiae*

*Streptococcus agalactiae*, which is also known as group B *Streptococcus* (GBS), has ten known serotypes (Ia, Ib, and II-IX) based on variant capsular polysaccharides [21]. In addition to its conserved genome region, its variable islands often harbour virulence genes responsible for the serious infectious disease caused by GBS in pregnant or postpartum women and their infants [22–24] as well as animals such as fish and cows [25, 26]. Because of the variation in its structural genomic framework, GBS was one of the first species to be studied in the fields of pan-genomics and comparative genomics [27]. In this study, 51 published GBS genomes from the NCBI database, including 28 complete genomes and 23 draft genomes, were collected for the modified GTN analysis and used to study phylogenetics at the gene and gene family levels as a demonstration of the new analytical approach.

## Results

### GTN performance

For the complete genome group, the GTN analysis with the BLAST+MCL assignment method took approximately 25.3 h with 4 threads in BLASTP alignment and 11.6 h for the analysis using SNP methods (panX, mafft, and RAxML). For the group with 46 complete and draft genomes, the GTN required an additional 6.3 h for common synteny block detection.

Roary is a pan-genome pipeline that can rapidly acquire protein clusters [28]. We enabled the GTN by using the Roary result as the GTN input to obtain the distance file (nwk) for approximately 110 min. Similarly, Roary performed gene cluster assignment by using BLAST and MCL. The difference between Roary and this version of the GTN is that Roary only aligns the protein sequences from the genomes to themselves, while the GTN additionally aligns the GBS proteins to the COG database (approximately 190,000 protein sequences). This is the main factor responsible for the lower performance of the GTN. The first version of the GTN only aligns protein sequences to COGs by using BLASTP to perform gene family assignment, so it requires less time to run (Tab. 1).

**Table 1:** Time required to perform in different methods

method	time
GTN (BLAST+MCL)	25.3 h
GTN (CD-HIT+DIAMOND)	30 min
GTN (Roary input)	110 min
SNP	11.6 h
First version of GTN	50 min

The tools in brackets represent different methods for performing gene family assignment

To solve this problem, we developed the CD-HIT+DIAMOND method to assign gene families instead of applying the BLAST+MCL method. With this optimization, the GTN only required 30 min for running. However, the resolution of the phylogenetic tree was inevitably lower than that obtained from the BLAST+MCL method. Thus, we used the BLAST+MCL assignment method to perform downstream analysis in this demonstration.

The main results obtained from the GTN are as follows: an evolutionary distance file (nwk) with 1000 bootstrap replicates, based on which the phylogenetic tree can be built; information on genes at unique node connections, which includes the unique node connections, the genes at these unique node connections, and the gene id in a GFF file (these genes differentiate the gene order); and the relative DD values of gene families, which are used to evaluate the connection-changing tendency of the genes in gene families.

### Exclusion of five genomes after validating genome completeness

The average length of the common synteny blocks of 51 genomes was 1,427.7 KB. After discarding each genome individually, the average length of the common synteny blocks of the remaining 50 genomes ranged from 1,428.2 KB to 1,449.5 KB (Additional file 2: Figure S1). There were sharp increases in the average length of the common synteny blocks by 21.8, 20.4, 18.5, and 14.9 KB after removing genomes GB00411, SA20–06, ATCC_13813, and GBS10, respectively, whereas when each of the other 46 genomes was removed, the average length of the common synteny blocks only increased from 0.59–9.01 KB (Additional file 2: Figure S1). Therefore, we set the threshold at 1% of the common synteny block length and eliminated these four genomes.

Since the COG database is a classical database for the functional annotation of gene classification, it can reflect the annotated gene coverage of all genes from a genome. The average proportion of the COG-annotated genes in all genomes was 72.9%. Two genomes, GBS10 and MC632, showed considerably lower COG-annotated gene proportions of 62.4 and 63.4%, respectively, which were obviously lower than those of the others as shown in Additional file 2: Figure S2.

Therefore, five genomes with low completeness, GB00411, SA20–06, ATCC_13813, GBS10, and MC632, were excluded from the subsequent analysis. A total of 46 complete and draft genomes with proper completeness were finally obtained. The average length of the synteny blocks of 46 of these genomes was 1,507.12 KB. Hence, the quantitative analysis method developed in this work is expected to have potential applications for evaluating the completeness of a genomic sequence for comparative genomic research.

### Summary of cluster assignment and COG classification

Among the 27 complete genomes of GBS, 93.0–99.9% (98.7% on average) of the genes were assigned to different gene orthologues by using orthoMCL software, and 56.1–64.4% (60.0% on average) of the genes were COG annotated by using the MCL algorithm in the GTN program (Additional file 2: Figure S3, Additional file 1: Table S1). Among the 46 complete and draft GBS genomes with proper completeness, 45.4–62.1% (53.2% on average) of the genes were located in the common synteny blocks, and 78.9–82.6% (81.2% on average) of the genes that were located were COG annotated by the GTN program (Additional file 2: Figure S4, Additional file 1: Table S2). Performing orthologous assignment using orthoMCL software resulted in a higher orthologous assignment efficiency. Nevertheless, the function of COG assignment embedded in the GTN tool exhibited a robust COG annotation capability, demonstrating its considerable potential as a user-friendly automatic COG assignment tool.

### Phylogenetic analysis of GBS genomes on the basis of the GTN

**Fig. 3:**
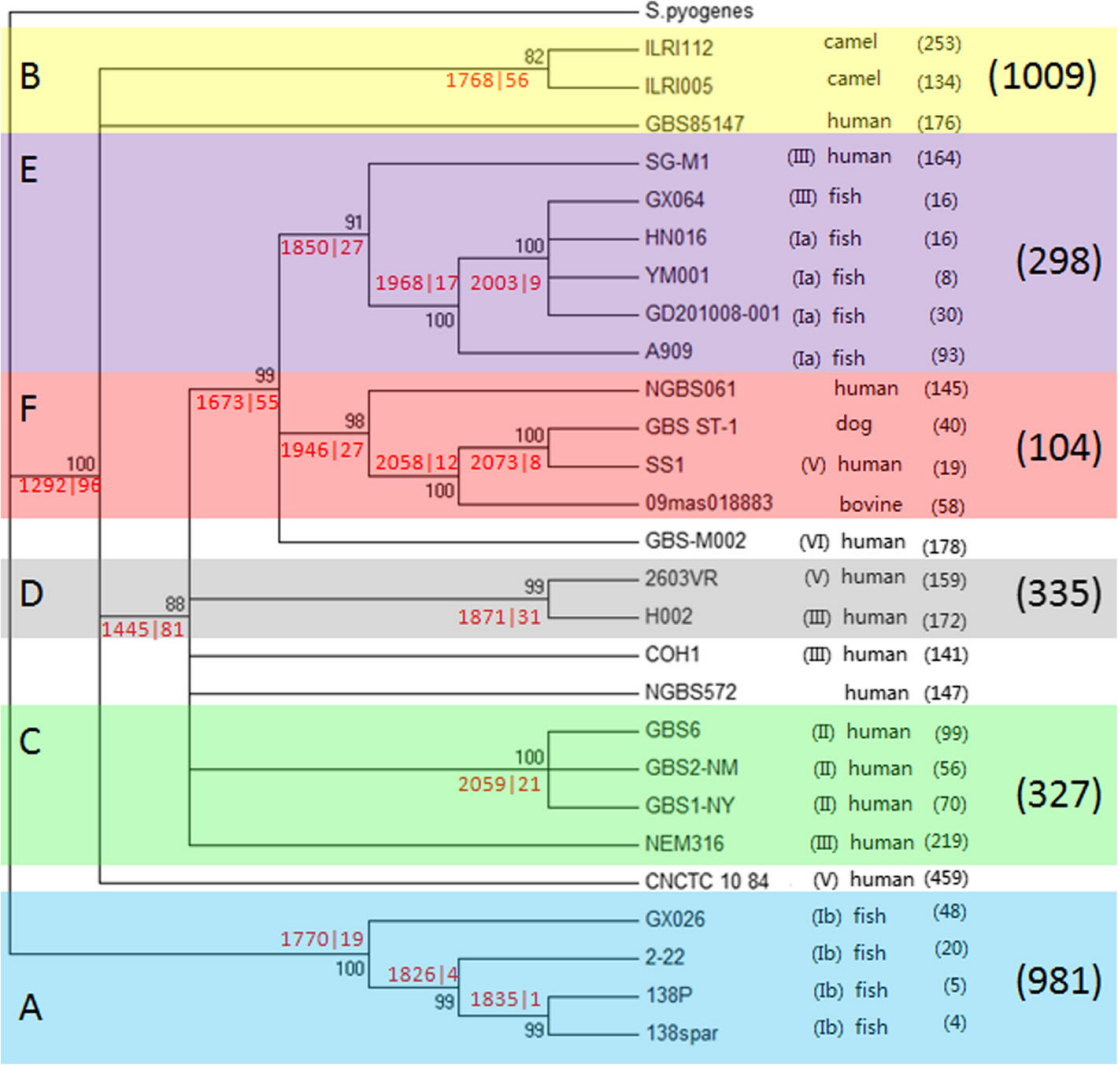
COG-based phylogenetic tree of the complete genome group. The number following each strain is the number of genes at unique node connections. The number following the six main clades is the number of genes at unique node connections that can be found in all genomes of the clade. The first red number before “|” in a cross is the length (KB) of the pieces that are connected based on the common node connections in the genomes of the clade. The second red number is the number of pieces

Similar phylogenetic trees were obtained from the phylogenetic analysis of the complete genome group on the basis of the COG (Fig. 3) and orthoMCL assignment results (Additional file 2: Figure S5), which both suggested that most of the 27 complete genomes of GBS may belong to six groups. In both phylogenetic trees, the GBS strains of serotypes Ia, Ib, and II were clustered into one group. Four strains could not be allocated to a certain subgroup, including the serotype VI strain GBS-M002, which was located close to clades E and F. GBS-M002 was originally clustered in clade F. A bootstrap test showed that the bootstrap value of this bifurcation was < 80, which is the cut-off threshold value, suggesting that it is unreliable. Thus, GBS-M002 was assigned as being parallel to clade F. The bootstrap values of the orthoMCL-based phylogenetic tree were higher than those of the COG-based tree. Fewer paraphylies were observed in the orthoMCL-based phylogenetic tree. However, given that genes can be classified into COG function categories by using the COG assignment function embedded in the GTN tool directly, the COG-based phylogenetic result was used to analyze the functional genes showing an altered gene order.

Random gene order permutations were set for each genome. Then, we used these random gene order permutations to build another phylogenetic tree by using the GTN (Additional file 2: Figure S6). The resulting constitutions of the tree were chaotic, indicating that phylogenetics cannot be assumed on the basis of an incorrect gene order.

The GTN phylogenetic tree of the group of 46 complete and draft genomes that was constructed with the COG gene family assignments confirmed the compatibility of the complete genomes with the draft genomes. According to this phylogenetic tree, five draft genomes were clustered in clade C, while two, four, and four draft genomes were clustered in clades D, E, and F, respectively (Additional file 2: Figure S7). The results showed that all serotype Ia GBS strains isolated from fish were clustered in clade E.

### Genome topology information provided via phylogenetic analysis by using the GTN method compared with that obtained by using the SNP method

Most of the constitutions of the clades in the phylogenetic tree (Additional file 2: Figure S8) obtained on the basis of SNPs by using the panX, mafft, and RAxML tools, were similar to those in our COG-based tree and orthoMCL-based tree, except for the position of genomes CNCTC_10_84 and NEM316. The SNP-based tree clearly showed that genome GBS-M002 could be clustered into clade F, and the bootstrap values of clade B were higher than those in the GTN-based tree. The COG-based tree exhibited the most paraphylies, and the orthoMCL-based tree presented the fewest. Compared to the SNP-based tree, the COG-based tree and the orthoMCL-based tree from the complete genome group distinguished the clade consisting of genomes 09mas018883, SS1, and GBS_ST-1 and the separation of genomes GX026 and 2–22 with a bootstrap value > 99 (red clades in Additional file 2: Figure S9).

According to the formulas in Fig. 1, the theoretical basis of the GTN is that different gene orders in genomes affect the differentiation within at phylogenetic tree. Hence, the user can extract all genes at the unique node connections, which results in a unique gene order to study in relation to why the genomes or clades are differentiated. As a demonstration of the methodology, we extracted the genes at the unique connections in six main clades and classified them according to functional categories to determine the functional genes that mainly affect their differentiation. These genes and annotations were provided by the GTN as results (genes_in_unique_connection.txt).

At each bifurcation in the COG-based tree, the black number represents the bootstrap evaluation value of the clade, while the first red number represents the average genomic fragment length (in KB) linked by the genes involved in the gene connection relationship. This relationship was shared by all the genomes of the clade according to the GFF file. These fragments can also be considered to be the common ancestor of the genomes. The second red number represents the average number of these fragments in each genome of the clade.

Clade F was used as an example to investigate the gene connection situation in every genome in this clade. The genes in the connection relationship shared by GBS_ST-1 and SS1 could be connected to 10 fragments ranging from 4.9 KB to 525.6 KB in GBS_ST-1 and to 6 fragments ranging from 170.0 KB to 582.5 KB in SS1. The total lengths of the fragments in GBS_ST-1 and SS1 were 2071.4 and 2076.4 KB, respectively. We marked the average of the total length in one genome and the number of fragments (i.e., 2073|8 in the phylogenetic tree in Fig. 3). The gene connections unique to each genome were excluded from these fragments, and the phylogenetic tree indicated that 15 genes were present in SS1 and that 40 genes were present in GBS-ST1. When 09mas018883 was added to the clade, the average fragment length decreased to 2058 KB. When genome NGBS061 was added, the average fragment length decreased to 1946 KB. When draft genomes were added to the calculation, the number of fragments increased, and the average length decreased (Additional file 2: Figure S7) because the GTN only calculated the common synteny blocks.

We compared the serotype VI genome GBS-M002 with the average 1946 KB fragment of clade F and found that the VI serotype genome consisted of 178 genes in the unique gene connections. We calculated COG function statistics for these genes and found that, except for the “[S] function unknown” and “[R] general function prediction only” categories, the proportions of the genes were highest in the “[L] replication, recombination, and repair” and “[G] carbohydrate transport and metabolism” categories (Additional file 1: Table S3). Therefore, the evolutionary events that have occurred in these two gene families played a major role in the differentiation of serotype VI GBS and clade F from the phylogenetic tree.

The genes located at unique node connections from the six clades in the phylogenetic tree were extracted by using the same method as was used to determine the functional categories that differentiate the six clades. The genes at the unique node connections of the six GBS clades were classified into COG functional categories (Tab. 2). Except for the two unclear function categories of “[R] General function prediction only” and “[S] Function unknown”, these genes related to evolutionary events exhibited associations with “[G] Carbohydrate transport and metabolism,” “[L] Replication, recombination, and repair” and “[J] translation, ribosomal structure and biogenesis”. The pathway enrichment results from DAVID showed that these genes with differentiated gene orders were mainly associated with metabolic pathways in clades A, B, C, E, and F. A total of 44 genes in clade D were also enriched in metabolic pathways, but the corresponding *p*-value was > 0.05 (*p* = 0.068, Tab. 3). Clade B possessed the greatest number of genes at the unique node connections among the six clades. A total of 1009 genes belonged to 308 COG families, including 108 genes in the “[G] carbohydrate transport and metabolism” category and 70 genes in the “[E] amino acid transport and metabolism” category. For the 5 other clades, 104–981 genes were found at the unique node connections.

**Table 2:** COG functional classification of genes at the unique node connections of the six main clades

Function classification	clade A	clade B	clade C	clade D	clade E	clade F	total
[S] Function unknown	159	141	44	44	52	14	454
[R] General function prediction only	127	123	35	38	30	9	362
[G] Carbohydrate transport and metabolism	73	108	25	22	38	3	269
[L] Replication, recombination and repair	68	58	29	44	32	26	257
[J] Translation, ribosomal structure and biogenesis	82	58	16	28	18	6	208
[M] Cell wall/membrane/envelope biogenesis	58	62	34	32	12	6	204
[K] Transcription	65	54	22	33	19	5	198
[E] Amino acid transport and metabolism	66	70	13	16	16	5	186
[H] Coenzyme transport and metabolism	42	62	16	22	14	6	162
[P] Inorganic ion transport and metabolism	45	54	23	18	10	3	153
[F] Nucleotide transport and metabolism	53	46	9	4	12	3	127
[O] Posttranslational modification, protein turnover, chaperones	24	33	10	4	12	3	86
[U] Intracellular trafficking, secretion, and vesicular transport	20	33	14	4	4	3	78
[V] Defense mechanisms	26	20	5	10	9	5	75
[D] Cell cycle control, cell division, chromosome partitioning	14	21	17	8	4	4	68
[T] Signal transduction mechanisms	21	20	5	4	10	2	62
[C] Energy production and conversion	18	17	3	2	4	1	45
[I] Lipid transport and metabolism	14	18	5	2	2	0	41
[Q] Secondary metabolites biosynthesis, transport and catabolism	6	8	0	0	0	0	14
[N] Cell motility	0	3	2	0	0	0	5
total	981	1009	327	335	298	104

**Table 3:** Pathway enrichment of the genes at the unique node connections of the six main clades

clade	pathway	p-value	genes number
A	sag01100: Metabolic pathways	5.2E-06	197
	sag01110: Biosynthesis of secondary metabolites	0.0053	81
	sag00230: Purine metabolism	0.013	43
	sag00564: Glycerophospholipid metabolism	0.028	12
	sag00550: Peptidoglycan biosynthesis	0.031	22
	sag00561: Glycerolipid metabolism	0.045	13
	sag00680: Methane metabolism	0.045	14
B	sag01100: Metabolic pathways	0.0014	200
	sag01110: Biosynthesis of secondary metabolites	0.0073	89
	sag00564: Glycerophospholipid metabolism	0.0074	18
	sag03060: Protein export	0.015	18
	sag00052: Galactose metabolism	0.016	31
C	sag01100: Metabolic pathways	0.0014	48
	sag01110: Biosynthesis of secondary metabolites	0.0073	17
	sag00564: Glycerophospholipid metabolism	0.0074	1
	sag03060: Protein export	0.015	7
	sag00052: Galactose metabolism	0.016	5
D	sag01100: Metabolic pathways	0.067	44
E	sag01100: Metabolic pathways	0.015	50
F	sag01100: Metabolic pathways	0.021	13

We additionally used our own Perl scripts to extract all gene connections in each of the six clades to compare the connections with their parallel clades in determining the genes at the unique node connections. Under this method, a clade was regarded as an entirety. The genes in the “[G] carbohydrate transport and metabolism”, “[L] replication, recombination, and repair”, and “[J] translation, ribosomal structure, and biogenesis” categories also showed high rates among the unique node connections (Additional file 1: Table S4, Additional file 2: Figure S10).

The relative DD value of a COG family indicates the change tendency of its adjacent genes. Thus, a high relative DD indicates that a COG family possesses a large number of different neighbouring genes in the genomes. In the COG-based tree, the relative DDs of 729 COG families were calculated. Only 7 COG families exhibited a relative DD value > 2 and an average DD value > 4 (Tab. 4), 6 of which were classified into “[L] replication, recombination, and repair”, while the function of COG4495 was unknown. When we set a random gene order, 202 COG families presented average DD values > 4 and relative DD values > 2, and all 7 COG families with a relative DD value > 2 in Tab. 5 ranked below the top 7.

**Table 4:**
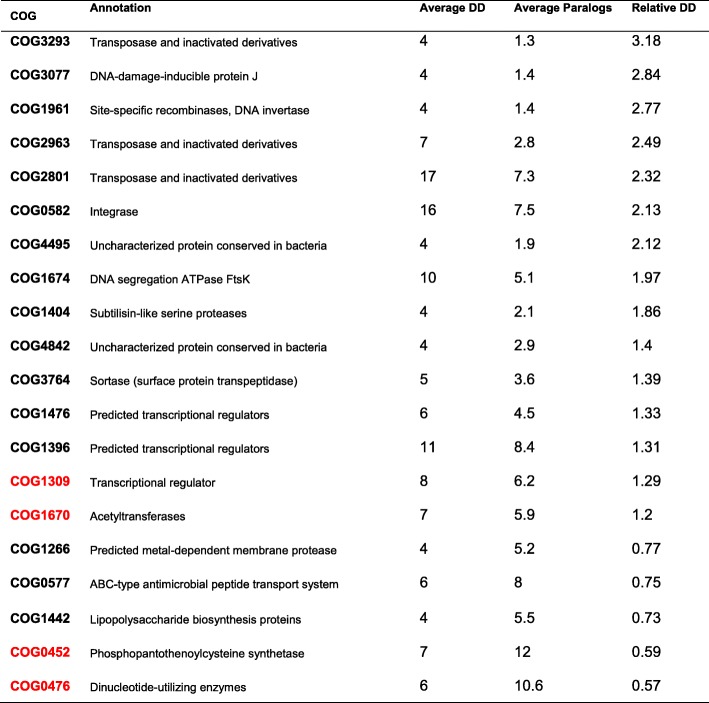
COG families with an average DD value > 4 in a complete genome group DD/str: average DD value. Para/str: average gene number for each genome. The COGs in red are included in both Tab. 4 and Tab. 5.

**Table 5:**
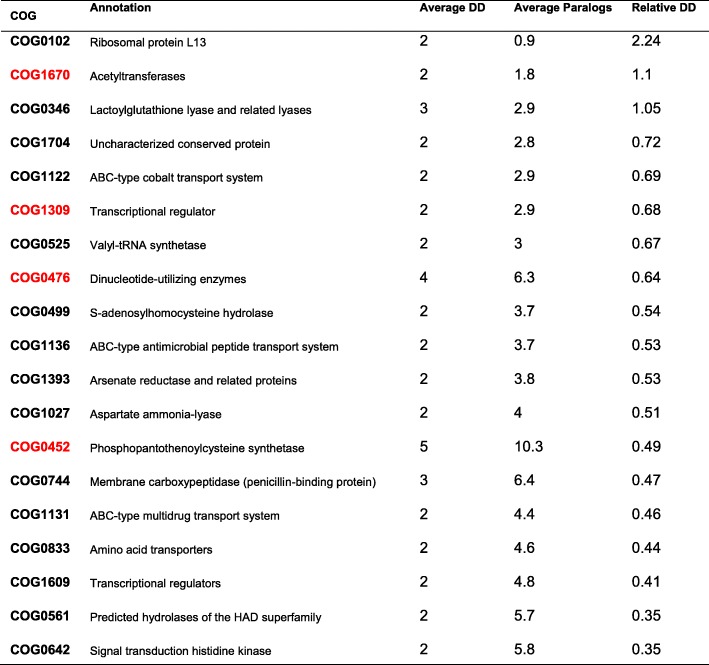
COG families with an average DD value > 2 in the complete and draft genome group DD/str: average DD value. Para/str: average gene number for each genome. The COGs in red are included in both Tab. 4 and Tab. 5

When the draft genomes were added to the input datasets, the GTN result reflected the evolutionary events in the common synteny blocks, which were the conserved regions of genomes. As a result, (Tab. 5), for the complete and draft genome groups, only one COG family exhibited a relative DD > 2: COG0102 (“[J] translation, ribosomal structure, and biogenesis”).

## Discussion

One of the improvements of our new GTN method in comparison with SNP analysis-based tools is that the GTN can provide detailed information on the genes at unique node connections, which are specifically responsible for the differences between genomes in the phylogenetic tree. Downstream analyzes such as functional enrichment analysis can then be performed on the basis of these genes. Pan-genome analysis tools may provide information on the gain or loss of core genes but may not focus on other genes or the copy number variations in the core genes, which will inevitably cause changes in the GTN.

Gene order represented by adjacent gene pairs is used in the GTN calculations, which may prove to be an effective approach for phylogenetic research. We list the differences between the GTN-based and SNP-based methods (panX, mafft, and RAxML) for generating the phylogenetic tree in Tab. 6. Although the applied methods were quite different, the general structures of the three trees were similar. Most of the strains were at the same locations, with some exceptions.

**Table 6:** Difference between the GTN and SNP-based methods

	GTN method	SNP method
Input file(s)	Fna, faa and gff format files	Gbk format file
Calculation region	Whole genome or common synteny block	Single-copy core genes
Evolutionary evidence	Gene order	SNP
Method for phylogenetic tree	Neighbour-joining	Maximum likelihood
What can be obtained	Neighbour-joining tree; genes at unique node connections; relative DD list; gene indel information; gene clusters (COG); common ancestor information	Maximum likelihood tree; core gene list; core gene alignment result; gene cluster

The SNP-based methods refer to the methods that we used in this study (panX, mafft and RAxML). The information on “genes at unique node connections” includes all genes at unique node connections. All these genes render an altered gene order, and they are evolutionary evidence of genomic evolutionary history (gene indels, duplications and recombination). The results shown in Tab. 2 are mainly based on these results. The information in the “relative DD list” includes all relative DD values of each COG family. The “gene indel” information includes genes in unique COG families or different copies of COG families. “Common ancestor information” includes the average length and number of fragments of a common ancestor; the red numbers in Fig. 3 were based on these results

Another potential advantage of the GTN method is that a large number of genomes can be included in the phylogenetic analysis, while the single-copy genes used under the SNP method may not be sufficient to provide a high resolution. There were 222 single-copy core genes among the 27 GBS strains used in the phylogenetic analysis, while approximately 60% (average of 1156 genes for each strain) of the COG-annotated genes and more than 90% (average of 1893 genes for each strain) of the orthologous genes were used in COG-based and orthologue-based GTN trees, respectively. We randomly selected 5 GBS strains three times, and the number of shared single-copy genes ranged from 600+ to 700+. When the strain number was increased to 10, 15, or 20, the number of single-copy genes was decreased to 400+, 300+, or 200+, respectively (Additional file 2: Figure S11).

We admit that SNP-based methods are still the gold standard for phylogenetic studies, and we suggest that the GTN method, which focuses on gene order, may provide a beneficial complement to improve the resolution of phylogenetic analysis for a number of close genomes. As an example, we considered three GBS strains (Additional file 2: Figure S9), SS1, GBS ST-1, and 09mas18883, and the bootstrap value did not meet the cut-off; therefore, the three strains were parallel in the SNP-based phylogenetic tree. We observed that the order of 56 orthologous genes of SS1 and 107 orthologous genes of GBS_ST-1 differed between the two strains and that 147 orthologous genes of 09mas18883 differed in order when strains SS1 and GBS_ST-1 were compared. The three strains could be clearly distinguished in the GTN-based trees, and we can directly determine which of these orthologous genes differ in order between the strains from the resulting calculations of the GTN. The genes whose order differed between two adjacent clades could also be extracted from the GTN results, and additional analysis such as functional enrichment analysis can be performed (Tab. 3).

Some genes were more mobile in that their relative DDs were higher than those of the others. Among the COG families with high relative DDs (Tab. 4) in the 27 complete GBS genomes, some belonged to the ‘Transposase and inactivated derivatives’ category, which consists of mobile genetic elements (MEGs) that may change position in the genome. In this group, a total of 345 genes were assigned to 10 COG families related to mobile genetic elements (1.1% of the total COG gene number). Mobile genetic elements have been well studied in relation to the evolution of genomes [29], providing substantial evidence for phylogenetic analysis. When the 19 draft genomes were introduced into the analysis, the range was narrowed from the whole genome to the common synteny blocks. Therefore, only 1507.12 KB of each genome was used by the GTN on average, and an average of 864 COG genes were located in these synteny blocks. We compared the blocks identified by the GTN and Mauve [30] in Additional file 1 Table. S5. It was reasonable that most of the transposase genes were not included in the common synteny blocks, which were assumed to be relatively highly conserved regions of the genome, and the relative DDs of the COG families declined as expected (Tab. 5). We compared the results of the present study with those of our previous work on *Mycobacterium tuberculosis* [14] and found that the GTNs of the two species were much different. Since the COG families of *M. tuberculosis* often have more paralogous members than those of GBS, the average DDs of *M. tuberculosis* were often higher, but the relative DDs were lower. Other than the transposase gene families, only one COG family, the COG1309 transcriptional regulator family (including the TetR/AcrR family transcriptional regulators and the dihydroxyacetone kinase transcriptional activator), occurred in the tables of the COG families with high relative DDs in both GBS and *M. tuberculosis*. This indicates that the GTN analysis may reveal some features of certain bacteria.

To determine how the dataset impacts the obtained resolution, we compared the orthoMCL-based tree, COG-based tree, COG family-based tree with MEGs removed, and database of essential genes (DEG)-based tree in one figure. We found that the resolution was ranked from highest to lowest as follows: orthoMCL-based tree, COG-based tree, COG-based tree with MEGs removed, and DEG-based tree (Additional file 2: Figure S12). There were 1908 genes on average in each genome used to build the orthoMCL-based tree and 1160 genes on average in those used to the build COG-based tree. Although the gene order of DEGs is considered to be the most stable structure in the genome, only 317 genes in each genome were used to build the tree; as a result, this tree presented the most parallels. Because of this practice, we assume that the resolution of a tree is most related to the gene number used in the genome topology network calculation.

## Conclusion

The modified GTN offers more functions than the first version and gives evolutionary information that the SNP-based method cannot give. Four improvements are implemented in the new GTN. Draft genome data can be included in the calculations of the new GTN. When draft genomes are added, the phylogenetic tree and relative DD values can indicate the evolutionary events in the conserved genome sequences. MCL, which is used in many protein-clustering tools, is introduced in the new version. Bootstrap test results can also be used to evaluate the robustness of each bifurcation. The information on the genes at unique node connections can explain the gene and clade differentiation in a phylogenetic tree. This GTN version may provide new insight into bacterial phylogenetics.

## Methods

### Data collection

All GBS genome data (at the complete genome, chromosome, and scaffold levels) were downloaded from the NCBI genome database in January 2016 (Additional file 1 Table. S6). All of the data came from the same species. Technically speaking, the GTN can analyze multiple species, but this function was not demonstrated in the current study. Among these genomes, 28 complete genomes at the complete or chromosome level were found, and 23 draft genomes at only the scaffold level were found. Each genome should contain FASTA nucleic acid (FNA), FASTA amino acid (FAA), and GFF files.

We set up the following two groups to process the genomic data efficiently: the complete genome group contained only complete genomes, for which the whole genome sequences were analyzed by the GTN; the other group contained all 51 complete and draft genomes, and every genome in this group was aligned to each other using the nucmer program (with default parameters) from MUMmer (version 3.23). The alignment results were intersected to obtain the regions in the genome that could be aligned to other genomes only once. We defined the intersecting regions as the common synteny blocks of this genome. The GTN only analyzed the common synteny blocks.

We also used Mauve (build date Feb 132,015, with default parameters), which is a multiple genome alignment tool, to find the conserved genomic sequences in the GBS genomes for comparison.

### Genomic data filtration

The common synteny blocks are the conserved genomic sequences that exist in all genomes. Thus, if a genome is incomplete or a considerable amount of its sequence is missing, then the common synteny blocks may be reduced considerably. Here, if the size of the common synteny blocks was increased by > 1% after discarding a single genome, then the genome was recognized as incomplete and unsuitable for this study. In the analysis of the complete and draft genome groups, 51 genomes were filtered primarily in terms of the average common synteny block length of the other genomes after removing one genome. Unqualified genomes were filtered out when the sizes of the common synteny blocks of other genomes were increased by > 1%. Since COG are the basic units for gene order, the greater the number of COG in a genome, the more accurate the calculations of the GTN will be. All protein sequences translated from 51 genomes were aligned to the COG database using BLASTP software to filter out genomes with low COG proportions.

### COG assignment and orthologous gene family construction

The function of COG assignment has been embedded in the GTN by introducing the MCL algorithm. After genomic filtration, the protein sequences of the genomes were integrated with the COG protein database into two FASTA files. Then, these two files were self-aligned using BLASTP [31] (version 2.2.26, parameters: -e < 1e-5 –m 9). The resulting COG family was processed into clusters by using mcxdeblast from the MCL package (version 14–137, parameters: --m9 --line-mode = abc --score = r) and the MCL algorithm (version 14–137, parameters: --abc) on the basis of the self-alignment results. The cluster with only one COG family was selected and considered as the functional annotation of the COG family.

OrthoMCL software (version 1.0, default parameters) was also used to obtain the orthologous families in the group of 27 complete genomes to evaluate the updated COG assignment function of the GTN tool.

### Phylogenetic analysis

When a gene family assignment result is completed, the GTN can use *f*_*1*_ in Fig. 1 to calculate the evolutionary distance and then build the NJ phylogenetic tree. In this study, three phylogenetic trees were built on the basis of the four different assignment results, as follows:

1. COG-based tree: a complete genome group tree constructed based on the COG family assignment results clustered by the MCL algorithm and embedded in the GTN. The function of the COG families was annotated.
2. OrthoMCL-based tree: a complete genome group tree built based on the orthoMCL software assignment results. The functions of the gene families were unclear.
3. DEG-based tree: The DEG database consists of essential genes [32]. We selected 317 essential genes of ‘*Streptococcus agalactiae* A909’ as representative sequences. All protein sequences from each genome were aligned to them by using BLASTP (version 2.2.26, parameters: -e 1e-5), and the aligned genes with the best hits were considered essential genes of this genome.
4. 46-genome tree: a tree consisting of 46 genomes obtained after genomic filtration with the COG gene families assigned by MCL.

The GTN used these gene family assignment results to construct topology networks and then used *f*_*1*_–*f*_3_ in the first GTN version [14] to calculate the evolutionary distance, obtain a distance matrix, and define unfixed genes. The R package ape (version 2.8, default parameter) [33] was applied to produce the distance matrix result by using the NJ algorithm with 1000 bootstrap replicates in the GTN. MEGA software (version 5.05, bootstrap cutoff < 80) [34] was used to derive a consensus of the bootstrap results (nwk file) and to then draw phylogenetic trees on the basis of the nwk file. The cut-off value was set to 80, and *Streptococcus pyogenes* was used as the out-group in the complete genome group.

We set a random gene order permutation for each GFF file and then built another phylogenetic tree as a null tree. Relative DD information was also obtained by the GTN.

To compare the phylogenetic trees calculated by the GTN, we used panX (version 1.5.1, default parameter) to identify the single-copy core genes of the complete genome group. Considering that panX lacks a bootstrap parameter, we aligned these genes by using mafft [35] (version 6.864b, default parameters) and then built a maximum likelihood phylogenetic tree by using RAxML (version 8.2.11, parameters: -e 1e-5 -p 12345 -# 1000 -m GTRGAMMA) with 1000 bootstrap replicates. MEGA software was also used to derive a consensus of the bootstrap results and draw the phylogenetic tree.

To optimize the running time of the GTN, we developed an alternative method for performing gene family assignment to the BLAST+MCL method. The genes were clustered by using CD-HIT [36], and the representative sequence of each cluster chosen by CH-HIT (version 4.8.1, parameters: -c 0.9 -g 1 -d 60 -M 0) was then aligned to the COG database by using DIAMOND (version 0.9.24.125, parameter: -sensitive) [37]; the best COG hit was considered as the functional annotation for this gene family.

### Adjacent gene analysis

In this improved version, the GTN provides information on every unique node connection in the genome (or clade) to its reference genome (or clade); these data include the gene id in the GFF file of gene production, gene function, and detailed connections. For a clade with more than one genome, the node connections existing in all genomes of the clade are compared to the node connections existing in all genomes of the parallel clade. The genes belonging to these nodes are also identified based on the GFF file.

### KEGG pathway enrichment

Since not all of the genes of GBS are recorded in the DAVID database (https://david.ncifcrf.gov/) [38]. The genes located at the unique connections in the six main clades were aligned against the proteins from GBS strain 2603 by using BLASTP (−e 1e-5), and the best hits ranked first in the alignment scores of each BLASTP alignment were considered to reflect the genes recorded in DAVID. These reflections were enriched using the DAVID database. These functions were lacking in the GTN tool.

## Supplementary information

**Additional file 1: Table S1.** Gene families used in the complete genome group. **Table S2.** Gene families used in the complete and draft genome group. **Table S3.** COG functional classification of the genes at the unique node connections in GBS-M002 against clade F. **Table S4.** All gene connections in the six clades for comparison with their parallel clades to determine unique node connections. The genes at the unique node connections were classified into COG functional categories. **Table S5.** Comparison of conserved genomic region detection between the GTN and Mauve. The ‘average length (KB) in a genome’ represents the average length of the conserved genomic regions in each genome. The ‘fragments in a genome’ represent the number of conserved fragments in each genome. **Table S6.** Information on 51 genomes downloaded from the NCBI database.

**Additional file 2: Figure S1.** Common synteny block filtration. **Figure S2.** COG gene percentage filtration. **Figure S3.** Numbers of genes with protein products and COG- and orthoMCL-annotated genes in the complete genome group. **Figure S4.** Numbers of genes with protein products, genes located in common synteny block areas, and COG-annotated genes in the complete and draft genome groups. **Figure S5.** Phylogenetic tree of the complete genome group based on the orthoMCL results. **Figure S6.** Phylogenetic tree built by random gene order permutation. **Figure S7.** Phylogenetic tree of the complete and draft genome groups on the basis of the COG result. **Figure S8.** Phylogenetic tree based on the SNP method obtained by using panX, mafft, and RAxML. **Figure S9.** Comparison between GTN- and SNP-based trees. **Figure S10.** Phylogenetic tree of the complete genome group based on the COG results with different methods of gene connection recognition in the clades. **Figure S11.** Number of single-copy core genes in each genome when we randomly select 5, 10, 15, 20 or 25 GBS genomes to perform pan-genome analysis with an out-group by using panX. **Figure S12.** Comparison of phylogenetic trees based on four different datasets.

## Data Availability

The GBS genomes data were collected from NCBI genome database (https://www.ncbi.nlm.nih.gov/genome/genomes/186?). Other data generated or analyzed during this study were included in this published article and its supplementary information files.

## Abbreviations

COG: Cluster of Orthologous Groups of proteins
DD: Different Degree
DEG: database of essential genes
FAA: FASTA amino acid
FNA: FASTA nucleic acid
GBS: Group B *Streptococcus*
GFF: General feature format
GTN: Genome Topology Network
KEGG: Kyoto Encyclopedia of Genes and Genomes
MCL: Markov Cluster algorithm
NCBI: National Center for Biotechnology Information
NJ: Neighbour-Joining
SNP: Single Nucleotide Polymorphisms
