# Supplementary material for: Modification of the genome topology network and its application to the comparison of group B Streptococcus genomes
Source: BMC Genomics. 2019 Nov 21;20:886. doi: 10.1186/s12864-019-6234-8 (PMC6868693; doi:10.1186/s12864-019-6234-8)
Supplement: Supplementary file 2 — Additional file 2: Figure S1. Common synteny block filtration. Figure S2. COG gene percentage filtration. Figure S3. Numbers of genes with protein products and COG- and orthoMCL-annotated genes in the complete genome group. Figure S4. Numbers of genes with protein products, genes located in common synteny block areas, and COG-annotated genes in the complete and draft genome groups. Figure S5. Phylogenetic tree of the complete genome group based on the orthoMCL results. Figure S6. Phylogenetic tree built by random gene order permutation. Figure S7. Phylogenetic tree of the complete and draft genome groups on the basis of the COG result. Figure S8. Phylogenetic tree based on the SNP method obtained by using panX, mafft, and RAxML. Figure S9. Comparison between GTN- and SNP-based trees. Figure S10. Phylogenetic tree of the complete genome group based on the COG results with different methods of gene connection recognition in the clades. Figure S11. Number of single-copy core genes in each genome when we randomly select 5, 10, 15, 20 or 25 GBS genomes to perform pan-genome analysis with an out-group by using panX. Figure S12. Comparison of phylogenetic trees based on four different datasets. [file 12864_2019_6234_MOESM2_ESM.docx]

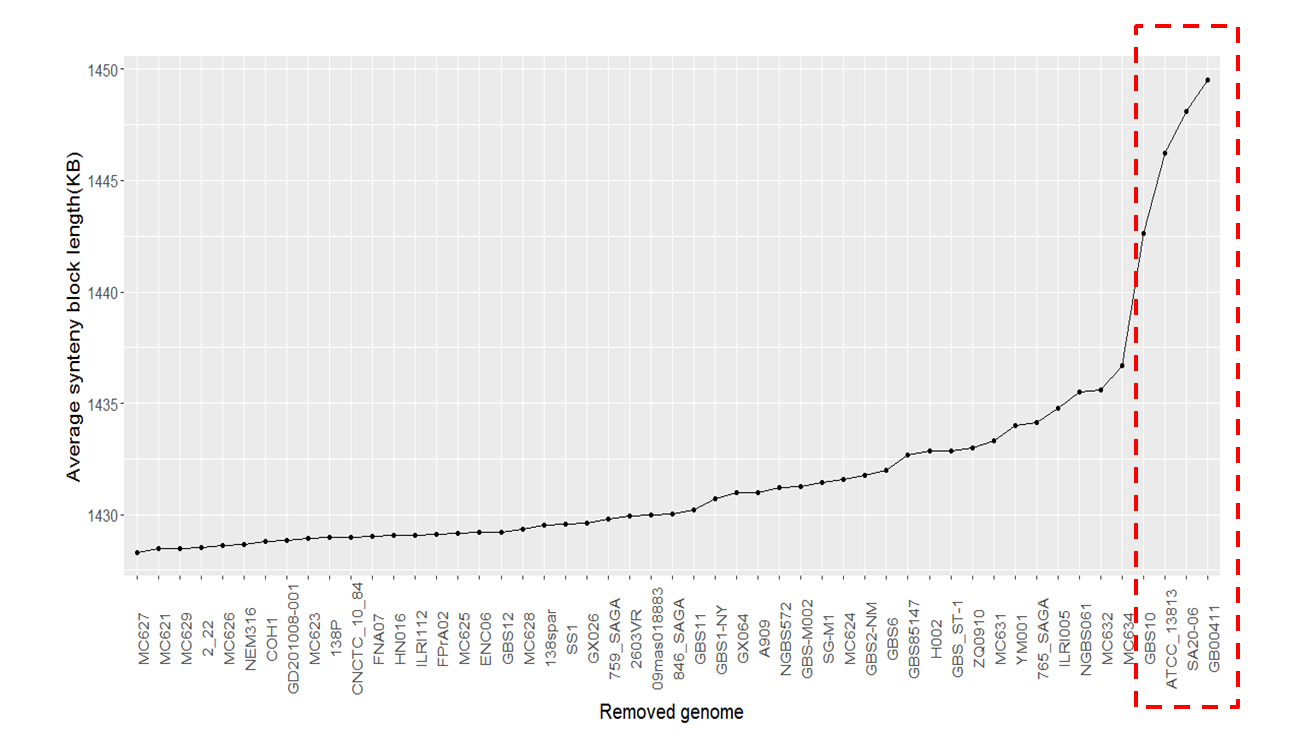


**Figure S1. Common synteny block filtration.** Average length calculation for the common synteny blocks of other strains with one genome discarded in the X-axis.


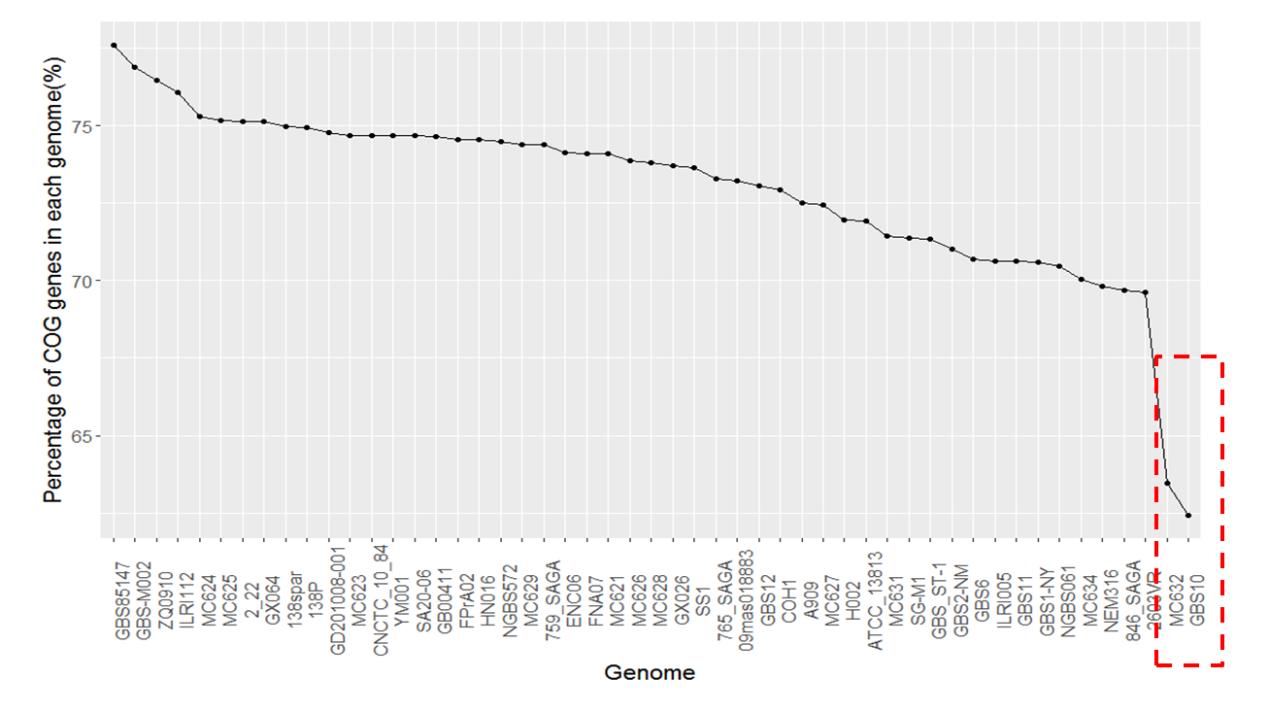


**Figure S2. COG gene percentage filtration.** The Y-axis represents the COG gene percentage in each GBS strain genome.


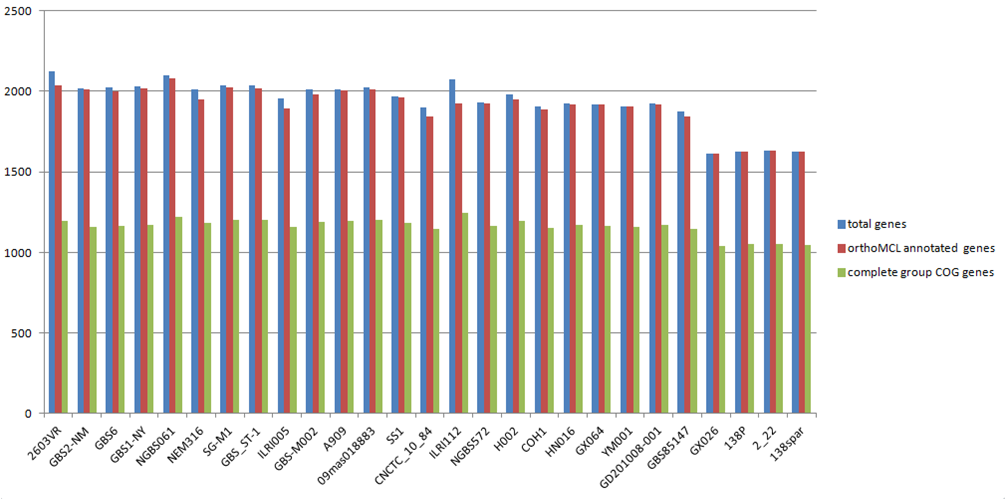


**Figure S3. Numbers of genes with protein products and COG- and orthoMCL-annotated genes in the complete genome group.** An average of approximately 98.7% of the genes in the 27 complete genomes were classified into different gene orthologue groups by using orthoMCL, and an average of nearly 60.0% of the genes in the 27 complete genomes were COG annotated by using MCL in the GTN program.


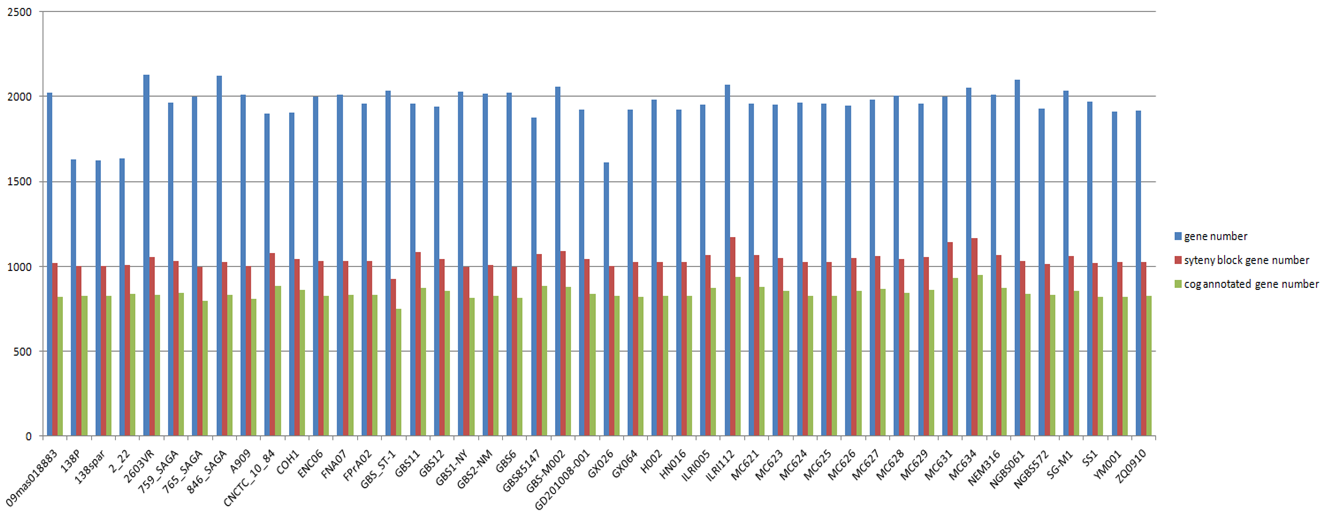


**Figure S4. Numbers of genes with protein products, genes located in common synteny block areas, and COG-annotated genes in the complete and draft genome groups.** An average of approximately 53.2% of the genes in the complete and draft genomes were located in common synteny block regions, and an average of approximately 81.2% of the genes located in common synteny block regions were COG annotated by MCL.


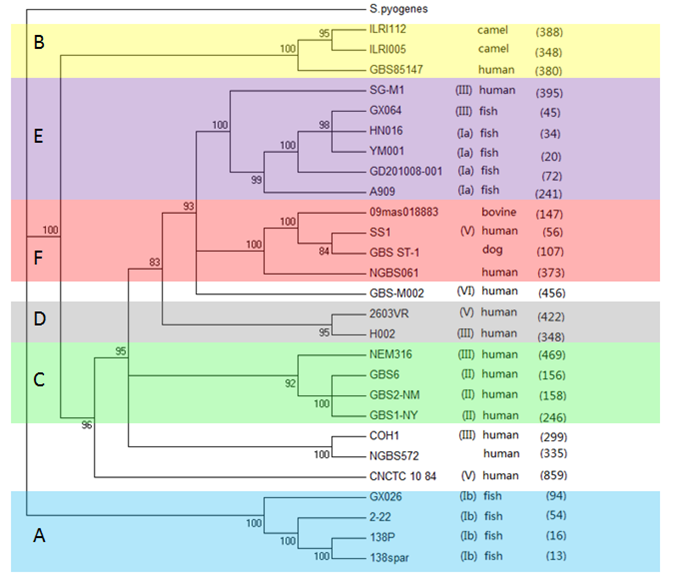


**Figure S5. Phylogenetic tree of the complete genome group based on the orthoMCL results.** The number following each strain shows the number of genes in the unique node connections.


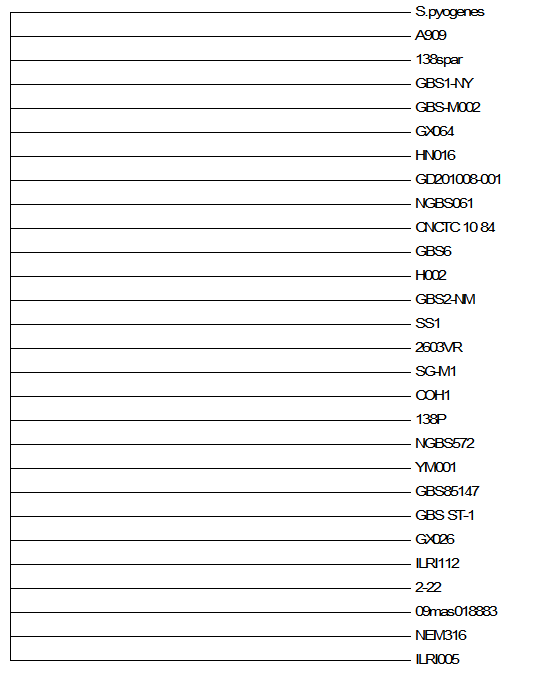


**Figure S6. Phylogenetic tree built by random gene order permutation.**


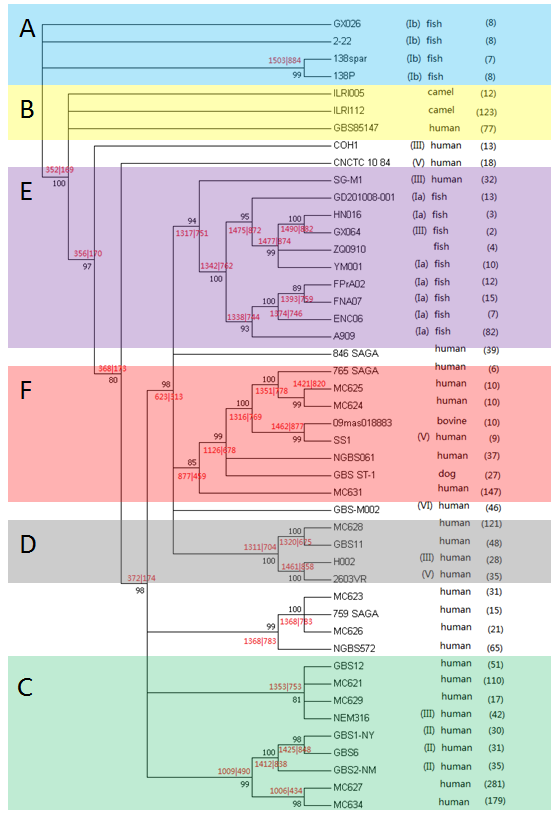


**Figure S7. Phylogenetic tree of the complete and draft genome groups on the basis of the COG result.** The number following each strain is the number of genes at the unique node connections. The first red number before “|” in a certain cross is the length (KB) of the pieces that are connected based on the common node connections in the clade genomes. The second red number is the number of pieces.

**
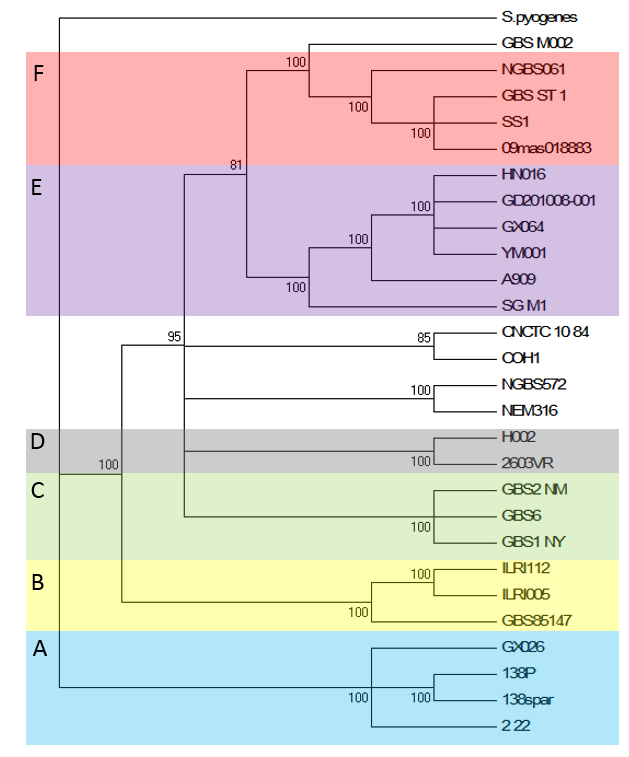
**

**Figure S8. Phylogenetic tree based on the SNP method obtained by using panX, mafft, and RAxML.**


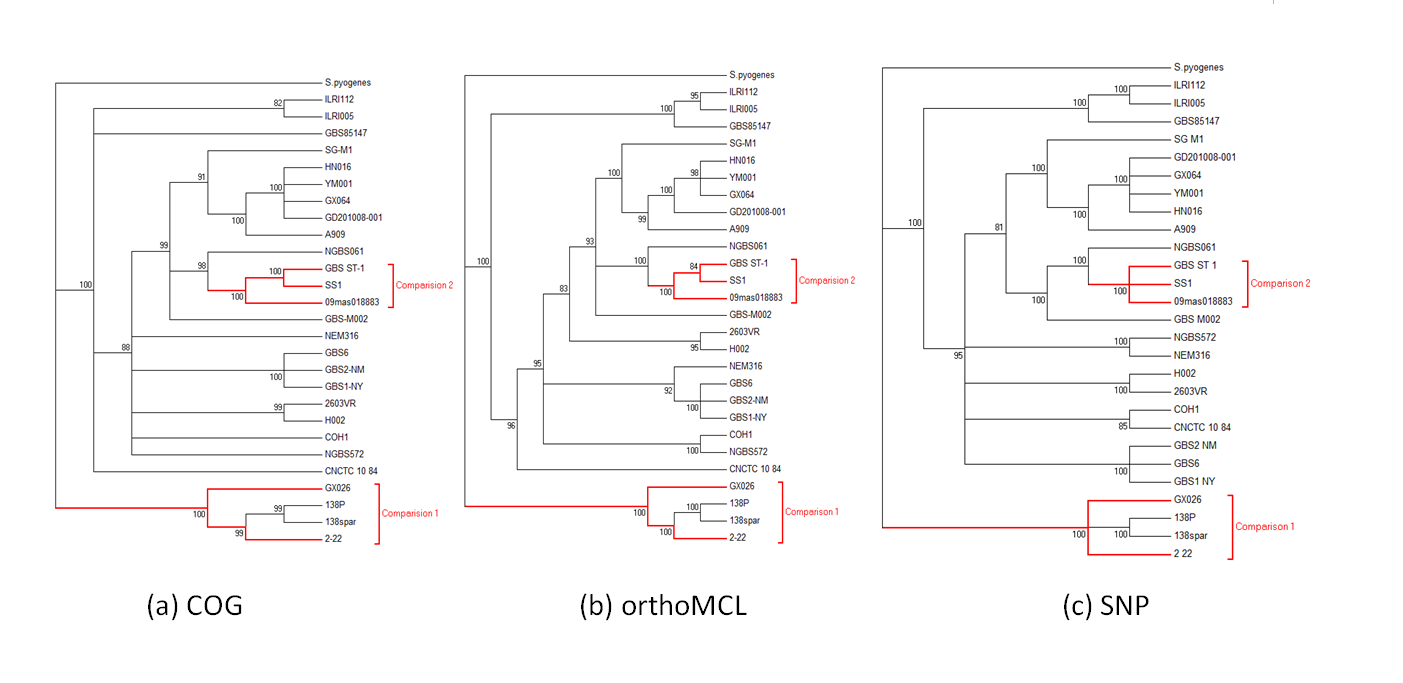


**Figure S9. Comparison between GTN- and SNP-based trees.** The tree in (a) is COG based, that in (b) is orthoMCL based, and that in (c) is SNP based. The COG-based tree shows more paraphylies, and the orthoMCL-based tree shows the least. Both GTN trees show a higher resolution for the red-coloured clades than does the SNP-based tree.


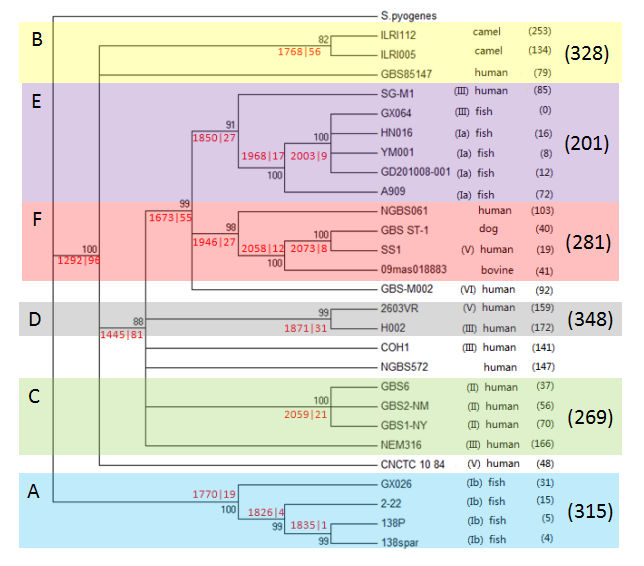


**Figure S10. Phylogenetic tree of the complete genome group based on the COG results with different methods of gene connection recognition in the clades.** The difference of this figure from Fig. 3 is that all gene connections in the query genome (or clade) are used (if the number of genomes is ≥2) for comparison with the parallel clade. As a result, the Arabic numerals in the brackets are different from those in Fig. 3.


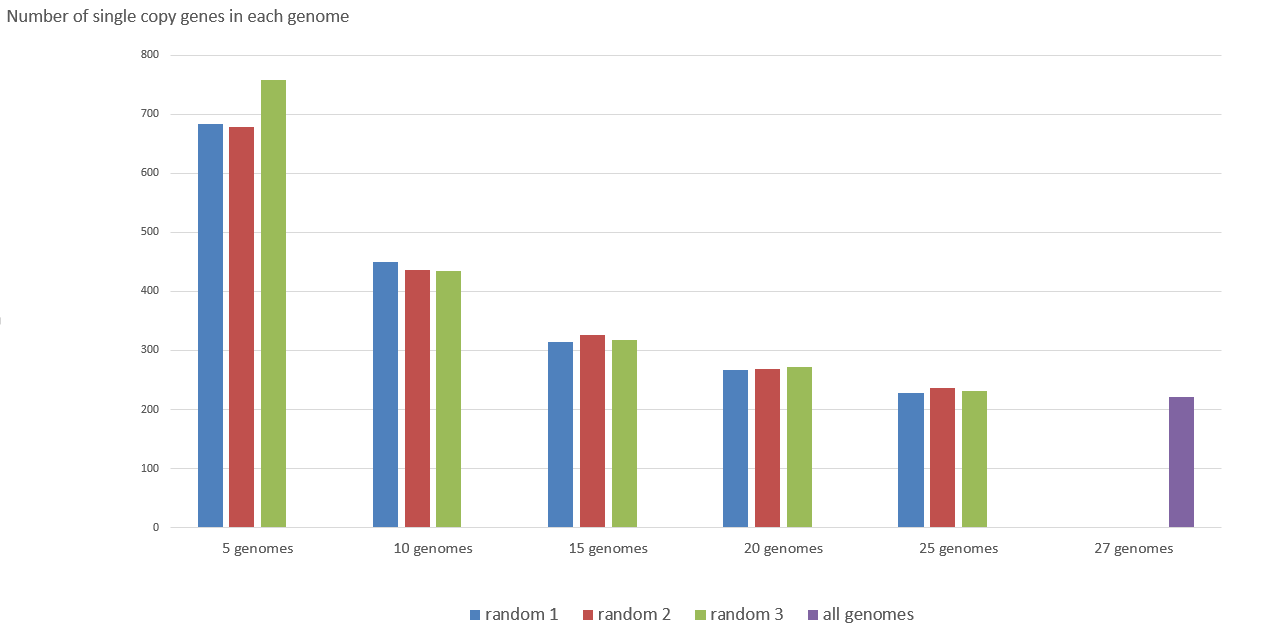


**Figure S11. Number of single-copy core genes in each genome when we randomly select 5, 10, 15, 20 or 25 GBS genomes to perform pan-genome analysis with an out-group by using panX.** Each group was randomly selected three times.


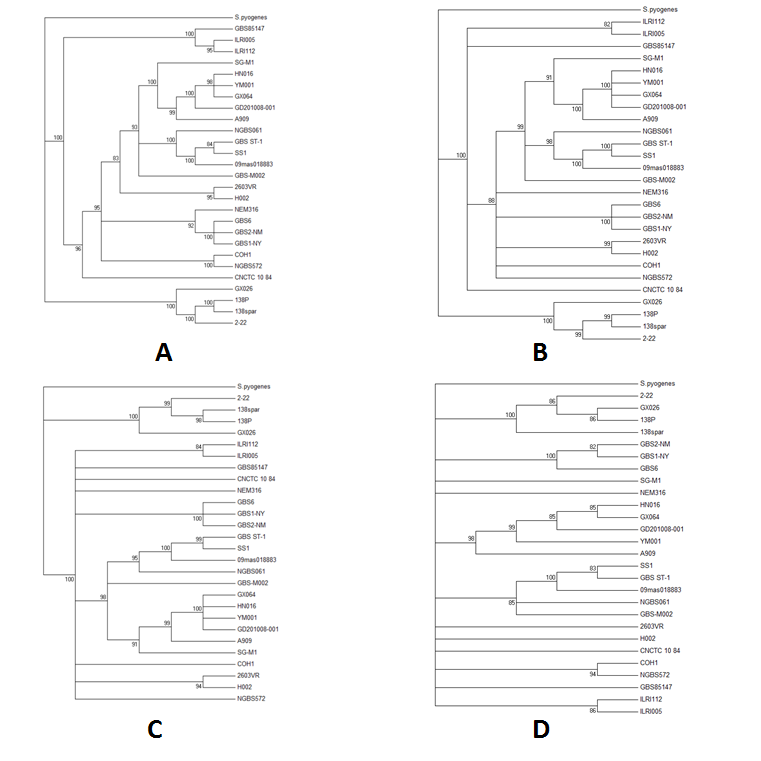


**Figure S12.** **Comparison of phylogenetic trees based on four different datasets.** The assignment in A. was based on the orthoMCL method. That in B. was based on the COG method. That in C. was based on the COG method with MEGs removed. That in D. was based on the DEG database.
